# Hyaluronic acid regulates a key redox control factor Nrf2 via phosphorylation of Akt in bovine articular chondrocytes

**DOI:** 10.1016/j.fob.2015.05.007

**Published:** 2015-05-29

**Authors:** Yuta Onodera, Takeshi Teramura, Toshiyuki Takehara, Kanji Fukuda

**Affiliations:** Institute of Advanced Clinical Medicine, Kindai University Faculty of Medicine, Osakasayama, Osaka, Japan

**Keywords:** ROS, reactive oxygen species, Nrf2, nuclear factor-erythroid-2-related factor 2, HA, hyaluronic acid, Hyaluronic acid, Reactive oxygen species (ROS), Nuclear factor-erythroid-2-related factor 2 (Nrf2), Chondrocytes

## Abstract

One important pharmacological function of hyaluronic acid (HA) in chondrocytes is reduction of cellular superoxide generation and accumulation. Here we demonstrated a relationship between HA supplementation and accumulation of Nuclear factor-erythroid-2-related factor 2 (Nrf2), which is a master transcription factor in cellular redox reactions, in cultured chondrocytes derived from bovine joint cartilage. In HA-treated chondrocytes, expression of Nrf2 and its downstream genes was upregulated. In HA-treated chondrocytes, Akt was phosphorylated, and inhibition of Akt activity or suppression of HA receptors CD44 and/or RHAMM with siRNAs prevented HA-mediated Nrf2 accumulation. Furthermore, Nrf2 siRNA inhibited the HA effect on antioxidant enzymes. These results show that HA might contribute to ROS reduction through Nrf2 regulation by activating Akt. Our study suggests a new mechanism for extracellular matrix (ECM)-mediated redox systems in chondrocytes.

## Highlights

• Hyaluronic acid (HA) has a pharmacological role for reduction of cellular superoxide.
• In HA-treated chondrocytes, expression of Nrf2 and its downstream genes was upregulated.
• Inhibition of Akt or suppression of HA receptors prevented HA-mediated Nrf2 accumulation.
• Nrf2 siRNA inhibited the HA effect on antioxidant enzymes.
• HA might contribute to ROS reduction through Nrf2 regulation by activating Akt.

## 1 Introduction

Articular cartilage is a unique tissue in which a single resident cell type, the chondrocyte exists in a specific environment that is avascular, aneural, alymphatic, but consist of abundant extracellular matrix (ECM) with a tissue specific pattern of proteoglycans (PGs), glycoproteins, collagens and hyaluronic acid (HA) [1–4]. This macromolecular assembly directly relates to maintenance of the chondrocyte phenotype [5–7]. HA is a major component of the cartilage ECM and synovial fluid [1,2,8]. It has been thought that the main biomechanical functions of HA are in lubrication and compressive stiffness of the tissue [9–11]. In osteoarthritis (OA)-affected joints, HA is modified through enzymatic degradation and oxidation, leading to compromised function [12,13]. In vitro studies have examined HA effects on cartilage tissue and cells [14,15]. For example, HA supplementation can promote normalization of the cartilage ECM environment, and have anti-inflammatory activities [16,17]. One of the important functions of HA is the control of oxidative damage [18]. We reported that HA reduces reactive oxygen species (ROS) production in mechanical stress-loaded bovine cartilage [19]. However, molecular mechanisms how HA participates in the control of ROS have not been elucidated.

Oxidative stress is caused by an imbalance between the production of ROS and biological detoxification systems, which are induced by inflammation, infection or excessive mechanical stresses [19–22]. Increased generation of ROS leads to acceleration of tissue injury, dysfunction and degradation by promoting cell death and ECM remodeling [23,24]. Under physiological conditions, the superoxide anions are detoxified by numerous antioxidants and phase II detoxifying enzymes such as catalase, superoxide dismutases (SODs), heme oxygenase-1 (HO-1), glutathione S-transferase, glutathione peroxidase, and thioredoxin. These enzymes are coordinated at transcription level by Nuclear factor-erythroid-2-related factor 2 (Nrf2) [25,26]. Nrf2 is constitutively expressed in all tissues of vertebrates and invertebrates. It plays an important role in maintaining cell survival and integrity by enhancing the protective capacity against oxidative stress [27]. In fact, disruption of Nrf2 in mice diminishes or abrogates the induction of these antioxidant genes, and contributes to susceptibility to neurodegenerative [28–30], cardiovascular [31,32], pulmonary [33,34], renal [35,36] and liver diseases [37,38].

By considering the above three facts that (1) ECM can alter chondrocyte gene expression, (2) HA can function as an antioxidant in cartilage and (3) protection against ROS is controlled by Nrf2 at the transcription level, we propose the hypothesis that the redox potential of HA may be related to enhancement of the Nrf2-mediated antioxidation potential in the chondrocyte. Here, we examine the regulation of Nrf2 expression in chondrocytes and effect of HA to Nrf2 expression using bovine articular chondrocytes in vitro.

## 2 Materials and methods

### 2.1 Culture and treatment of bovine primary articular chondrocytes

Full thickness articular cartilage tissues were collected from the condylar ridge of the metacarpophalangeal joints of freshly slaughtered calves about 10 months of age, which were donated from a local slaughterhouse. Chondrocytes were isolated from the articular cartilage by enzymatic digestion with 2 mg/ml of collagenase (Wako Pure Chemical Industries, Osaka, Japan) for 12 h at 37 °C. After filtration, cells were seeded in culture plates and cultured in 10% FCS supplemented αMEM at 37 °C and 5% CO_2_, 5% O_2_ for 48 h. In the experiments, the medium was changed to serum-free medium consisting of 0.5% bovine serum albumin (BSA, Sigma–Aldrich, St. Louis, MO, USA), DMEM/F12 (Life Technologies Inc., Carlsbad, CA, USA), 2 mM l-glutamine (Wako Pure Chemical Industries, Tokyo, Japan), 1% insulin-transferrin selenium (Life technologies) and 1% antibiotic/antimycotic solution (Life Technologies). Cells were cultured under these conditions for 24 h when the following reagents were added; 1 mM H_2_O_2_ (Wako Pure Chemical Industries), 10% hyaluronic acid (ARTZ, approx. 500,000–700,000 MW, Kaken Pharmaceutical Co., Ltd., Tokyo, Japan), 20 μM LY294002 (Sigma–Aldrich) and same amount of DMSO as a control of LY294002 (Sigma–Aldrich).

### 2.2 Measurement of intracellular reactive oxygen species (ROS)

Generation of the ROS was detected using CellROX® Green Reagent for oxidative stress detection (Life technologies). Bovine chondrocytes at passage 2 were seeded on 48-well plate and incubated with H_2_O_2_ with/without HA for 2 h. After washing twice with culture medium, the cells were treated with CellROX Green Reagent for 30 min at 37 °C following manufacturer’s instructions. Fluorescence intensity was analyzed by a Wallac ARVO MX 1420 multilabel counter (Perkin Elmer Japan, Kanagawa, Japan).

### 2.3 siRNA transfection

For the gene knock-down experiments, chondrocytes at passage 2 were seeded onto 48-well multiplates and cultured in the serum-free medium for 24 h. Then a mixture of three specific siRNAs (Table 1) was transfected into chondrocytes using Lipofectamine RNAiMAX Reagent (Life Technologies) according to the manufacturer’s protocol. Twelve hours later, the complexes were removed from the 48-well plates, cells were washed with PBS, and 400 μl fresh serum-free medium containing the reagents of interest was added to the plates. The controls treated with scrambled siRNA at the same concentration were analyzed in parallel.

### 2.4 Western blot analysis

After each treatment, cells were lysed with SDS buffer (4% SDS, 125 mM tris–HCl, 10% 2-mercaptoethanol, 0.01% bromophenol blue in 20% glycerol) then the total cell lysates were separated by SDS–PAGE and transferred to polyvinylidene difluoride (PVDF) membranes. (Hybond-P; Amersham Pharmacia Biotech, Buckinghamshire, UK). The blotted membranes were blocked at room temperature for 1 h with Block Ace (Dainippon Pharmaceutical, Osaka, Japan) and treated with following primary antibodies overnight at 4 °C; Phospho-p38 MAPK (Thr180/Tyr182) (D3F9) XP rabbit monoclonal antibody (Cell Signaling Technology, Danvers, MA, USA), p38 MAPK rabbit polyclonal antibody (Cell Signaling Technology), Phospho-p44/42 MAPK (ERK1/2) (Thr202/204) rabbit polyclonal antibody (Cell Signaling Technology), p44/42 MAPK (ERK1/2) (137F5) rabbit monoclonal antibody (Cell Signaling Technology), Phospho-Akt (Ser473) (D9E) XP rabbit monoclonal antibody (Cell Signaling Technology), Akt rabbit polyclonal antibody (Cell Signaling Technology), Keap1 (D6B12) rabbit monoclonal antibody (Cell Signaling Technology), Nrf2 (C-20) rabbit polyclonal antibody (Santa Cruz Biotechnology, Santa Cruz, CA, USA) and GAPDH mouse polyclonal antibody (Abnova, Taipei, Taiwan) followed by a peroxidase-conjugated anti-rabbit or mouse IgG antibody (Santa Cruz Biotechnology). Detection was realized by enhanced chemiluminescence with an ECL prime western blotting detection reagent (Amersham Pharmacia Biotech, Buckinghamshire, UK) and horseradish peroxidase (HRP)-conjugated secondary antibodies (all were purchased from Santa Cruz Biotechnology) corresponding to each primary antibody. The lumino-labeled membranes were analyzed by the CCD-based chemiluminescent analyzer LAS 4000 (GE Healthcare, Tokyo, Japan).

### 2.5 Quantitative RT-PCR (qRT-PCR) analysis

Total RNA was extracted with the TRIzol reagent (Invitrogen) and reverse transcribed with the High Capacity cDNA reverse transcription kit (Applied Biosystems, Foster City, CA, USA). The qRT-PCR with total cDNA was performed using Perfect real-time SYBR green II (Takara Bio, Inc., Shiga, Japan) with appropriate primer sets (Table 2) in the Thermal Cycler Dice® Real Time System (Takara Bio, Inc.) at 95 °C for 20 s followed by 40 cycles of 95 °C for 5 s, 60 °C for 30 s. To quantify the relative expression of each gene, the Ct (threshold cycle) values were normalized to an endogenous reference (ΔCt = Ct*_target_* − Ct*_Gapdh_*) and compared with a calibrator (control), using the ΔΔCt method (ΔΔCt = ΔCt_sample_ − ΔCt_calibrator_).

### 2.6 Statistical analysis

Significant difference was detected by Tukey–Kramer HSD test or Student’s *t*-test. A *P*-value of less than 0.05 was considered significant.

## 3 Results

**Fig. 1:**
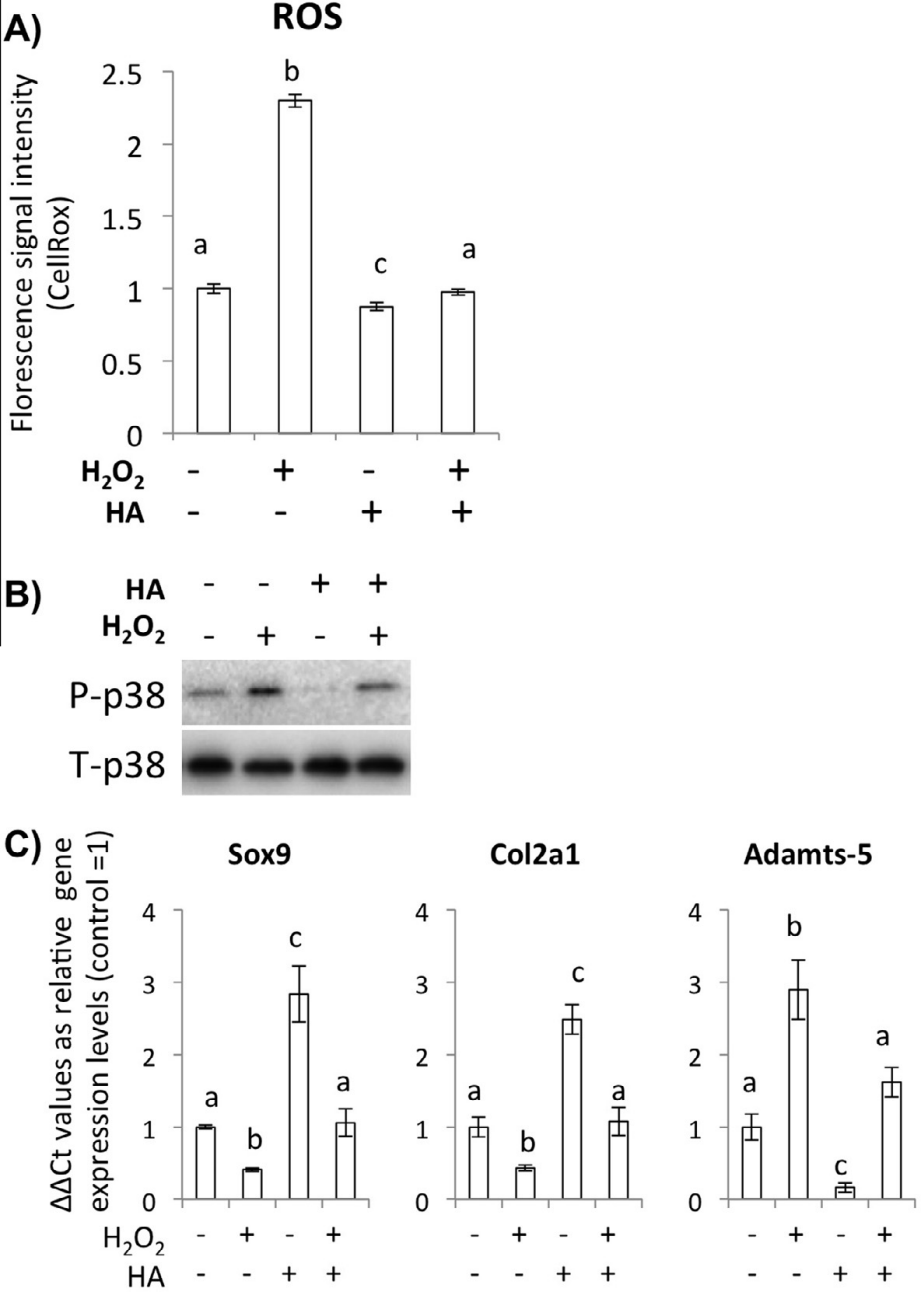
HA decreased ROS accumulation in H_2_O_2_ treated chondrocytes. (A) CellROX-based assay for observing ROS levels of the chondrocytes. HA addition decreases ROS levels detected as the CellROX fluorescence intensity. Bars show means ± SD of the results obtained by three independent experiments. An asterisk indicates significant difference at *P* < 0.05. (B) Western blot analysis for phosphorylation status of the redox-induced MAPK p38. (C) qRT-PCR analysis for the matrix related genes Sox9 and Col2A1 and catabolic gene Adamts-5. Bars show means ± SD of the results obtained by three independent experiments. Means with different letters are significantly different at *P* < 0.05.

As the first step of the study, we determined the antioxidant effect of HA in cultured bovine articular chondrocytes. To observe accumulation of ROS in the cells in response to hydrogen peroxide (H_2_O_2_) stimulation, we used CellROX Green Reagent that is a fluorogenic probe for measuring oxidative stress in live cells. By using CellROX, we could observe that H_2_O_2_ addition induced ROS accumulation in the chondrocytes, and this was inhibited by HA supplementation (Fig. 1A). ROS treatment decreased matrix anabolic factors Sox9 and Col2A1, while matrix degradation factor Adamts-5 was elevated. The addition of HA completely blocked the inhibitory effect of ROS on Sox9 and Col2A1 while significantly reducing Adamts-5. ROS also increased phosphorylation of p38 MAP kinase, which is an important indicator and mediator of oxidative stress, and this was reduced by HA (Fig. 1B and C). These results indicate that H_2_O_2_ induced ROS elevation is detrimental to matrix maintenance, and HA supplementation can function as an antioxidative factor to assuage the catabolic changes at gene expressions level in the cultured chondrocytes.

**Fig. 2:**
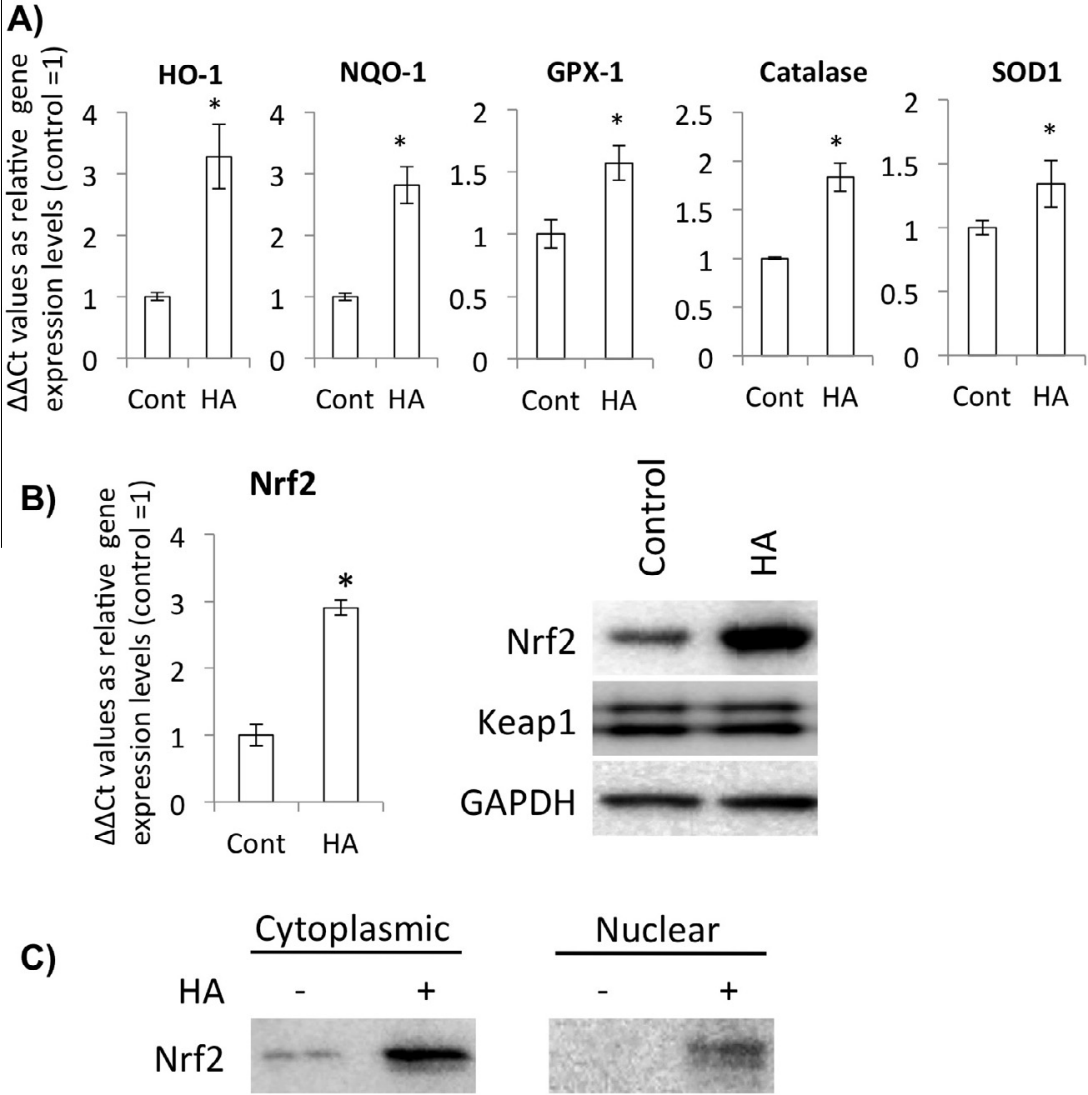
HA enhanced gene expression levels of the antioxidant and phase II detoxifying enzymes via increasing the Nrf2 expression level. (A) Quantitative RT-PCR analysis of gene expression levels of HO-1, NQO-1, GPX-1, Catalase and SOD1 in the HA treated/non-treated chondrocytes at 24 h of treatments. Bars show means ± SD of the results obtained by three independent experiments. Asterisks indicate significant differences at *P* < 0.05 compared with the untreated controls. (B) qRT-PCR and western blot analysis with the chondrocytes treated HA for 24 h. Bars show means ± SD of the results obtained by three independent experiments. An asterisk indicates significant difference at *P* < 0.05 with the untreated control. (C) Western blot analysis of the cytoplasmic and nuclear Nrf2 expression levels. Chondrocytes were treated with HA for 24 h.

Next, we examined whether the antioxidative effect of HA was exhibited through elevation of ROS neutralizing enzymes. After 24 h of HA treatment, we could observe increased HO-1, NQO-1, GPX-1 and catalase gene expression (Fig. 2A). Since these phase II detoxifying enzymes are controlled by a master transcription factor Nrf2, we analyzed Nrf2 expression levels both at gene and protein level. We could detect increased Nrf2 expression both at transcription and protein levels in response to HA (Fig. 2B). Increased Nrf2 protein was detected in both cytoplasmic and nuclear compartments (Fig. 2C). Expression level of Keap1, which is an important post-transcriptional negative regulator of Nrf2, did not change.

**Fig. 3:**
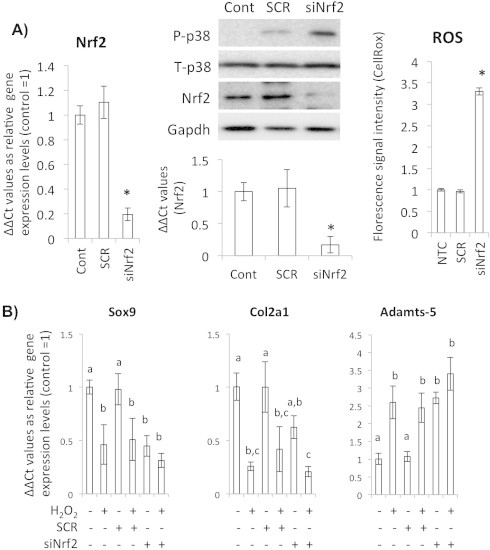
Nrf2 is a critical factor to scavenge ROS in chondrocytes. (A) Quantitative RT-PCR for gene expression levels of Nrf2 after siNrf2 treatment. Bars show means ± SD of the results obtained by three independent experiments. Asterisks indicate significant differences at *P* < 0.05 compared with the untreated controls (left). Western blot analysis for the siNrf2 treated chondrocytes to detect p38 phosphorylation as an intracellular ROS messenger (center, upper panel). Densitometric quantification of western blot for Nrf2 in the siNrf2 treated chondrocytes (center, lower panel). CellROX-based assay for observing ROS levels of the siNrf2-treated chondrocytes (right). Cont means non-treated control and SCR means scrambled siRNA treated cells. (B) Quantitative RT-PCR for gene expression levels of Sox9, Col2A1 and Adamts-5. SCR means scrambled siRNA treated cells. Bars show means ± SD of the results obtained by three independent experiments. Means with different letters are significantly different at *P* < 0.05.

Next, we examined if HA-induced increase in Nrf2 is actually linked to anti-oxidative stress potential of the cultured chondrocytes using specific siRNA oligonucleotides (siNrf2) which diminished Nrf2 mRNA to 20% of control cells that were treated with random RNA oligonucleotides (scramble, SCR). In the Nrf2-knocked-down chondrocytes, basal ROS level was elevated, and phosphorylation of p38 was induced (Fig. 3A). Furthermore, in the Nrf2-knocked-down chondrocytes, reduced Sox9 and Col2A1 and increased Adamts-5 mRNA levels were detected (Fig. 3B).

**Fig. 4:**
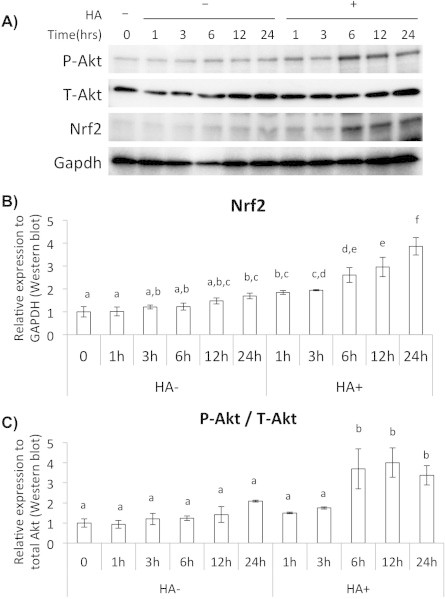
HA-induced Nrf2 accumulation in chondrocytes was mediated by enhancement of Akt phosphorylation. (A) Western blot analysis for the HA treated chondrocytes to observe Akt phosphorylation and Nrf2 accumulation at each time point after HA addition. (B) Densitometric quantification of western blot for Nrf2. Values were normalized by GAPDH. (C) Densitometric quantification of western blot for phosphorylated Akt. Values were normalized by total Akt.

**Fig. 5:**
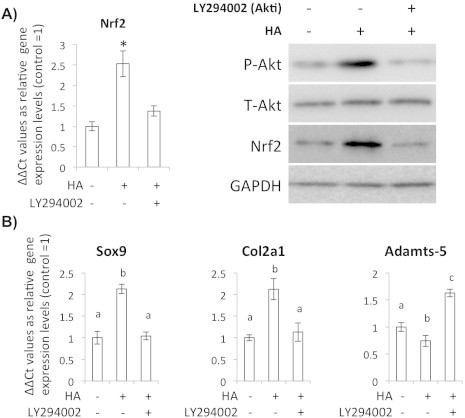
(A) qRT-PCR and western blot analysis for the chondrocytes treated with HA and Akt specific inhibitor LY294002 (Akti) for 24 h to observe Akt phosphorylation and Nrf2. Control (without LY294002) groups were treated with DMSO. Bars show means ± SD of the results obtained by three independent experiments. An asterisk indicates significant difference at *P* < 0.05 with the untreated control. (B) Quantitative RT-PCR observation for gene expression levels of Keap1, Sox9, Col2A1 and ADAMTS5 in the HA/LY294002 treated chondrocytes. Control (without LY294002) groups were treated with DMSO. Bars show means ± SD of the results obtained by three independent experiments. Means with different letters are significantly different at *P* < 0.05.

To elucidate mechanism of the HA-induced Nrf2 accumulation, we analyzed phosphorylation status of Akt, which is known as an upstream regulator of the Nrf2 stabilization. When we quantified changes of phosphorylated Akt and Nrf2 after HA addition to the chondrocytes, both proteins showed time-dependent increases (Fig. 4A–C). To confirm that the HA-induced Nrf2 accumulation is Akt-phosphorylation dependent, we added the Akt specific inhibitor LY294002 and performed western blot analysis. LY294002 treatment inhibited Akt phosphorylation without changing of the amount of total Akt protein, and it also diminished Nrf2 gene expression and protein accumulation (Fig. 5A). In the Akt inhibited condition, gene expressions of Sox9 and Col2A1 decreased, but the expression level of Adamts-5 increased (Fig. 5B).

**Fig. 6:**
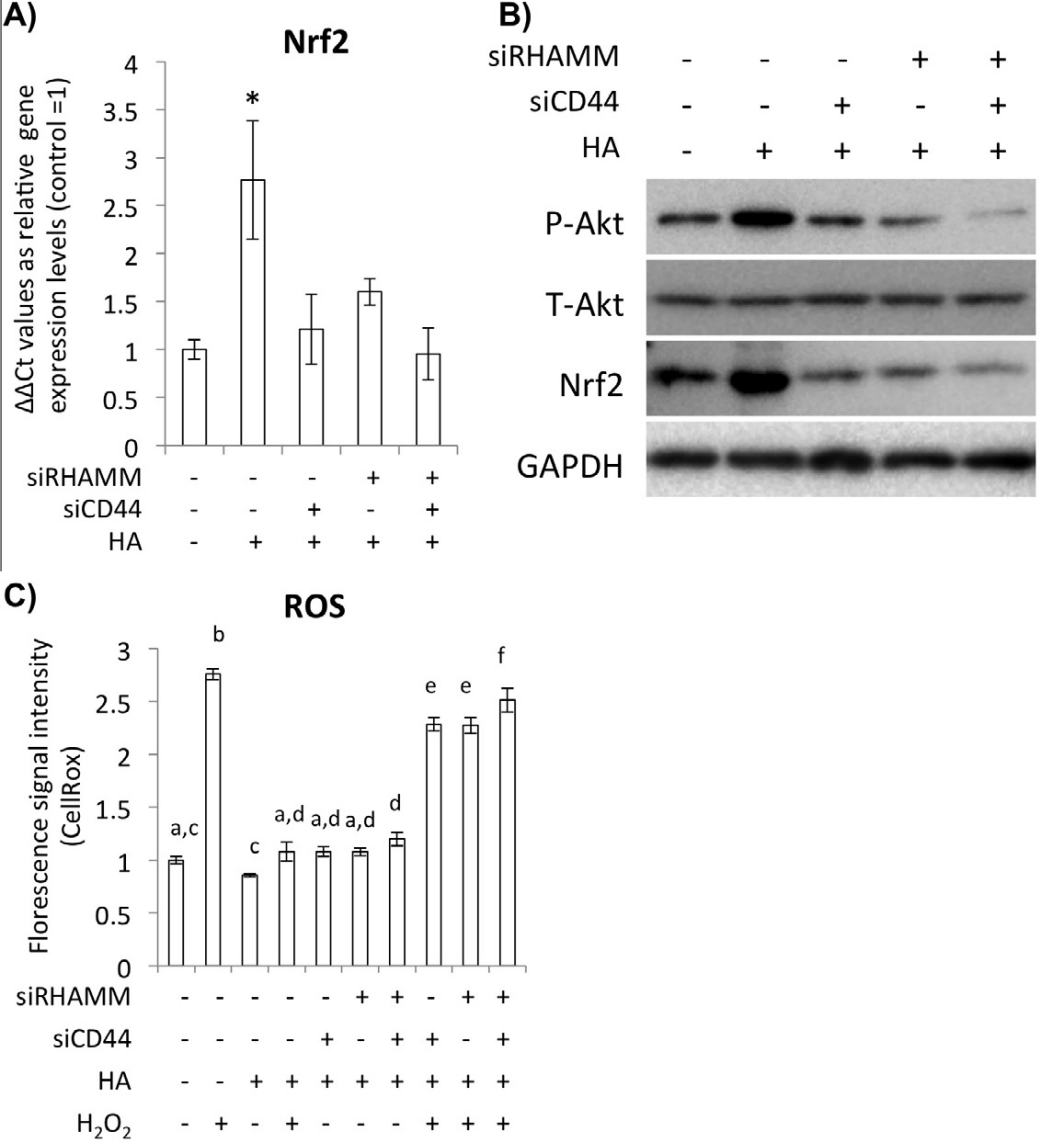
HA induced Akt phosphorylation and Nrf2 expression via HA receptors CD44 and RHAMM. (A) Quantitative RT-PCR for Nrf2 in the siCD44/siRHAMM treated chondrocytes at 24 h after HA treatment. Bars show means ± S.D of the results obtained by three independent experiments. Experimental group of siCD44 (−) and siRHAMM (−) was treated with scrambled sequence RNA as control. An asterisk indicates significant difference at *P* < 0.05 with the untreated control. (B) Western blot analysis for Akt phosphorylation and Nrf2 expression levels in the HA receptors CD44 and RHAMM-knocked-down chondrocytes after HA treatment. (C) Quantification of intracellular ROS by CellROX in the CD44 and RHAMM-knocked-down chondrocytes.

As the last part of the study, we determined whether the HA dependent regulation of Nrf2 expression occurred through interaction of HA with its specific receptors CD44 and RHAMM on the chondrocytes by inhibiting their expression. Introduction of the specific oligonucleotide towards CD44 and RHAMM (siCD44 and siRHAMM) decreased mRNA levels of the each gene. Importantly, HA-induced Akt phosphorylation, Nrf2 gene expression and Nrf2 protein accumulation were blocked in the CD44- and RHAMM-knocked-down chondrocytes (Fig. 6A and B). Furthermore, intra-cellular ROS was also increased in the CD44 and RHAMM-knocked-down cells (Fig. 6C). From these results, it could be concluded that the HA-induced Nrf2 accumulation in chondrocytes occurred through interaction of HA with its specific receptors CD44 and RHAMM.

## 4 Discussion

This study examined the hypothesis that the protective effects of HA in chondrocytes may be related to enhancement of the Nrf2-mediated antioxidation potential. Our results demonstrate an antioxidant effect of HA in cultured chondrocytes, and that HA supplementation results in the chondrocyte specific matrix-related gene expression. In a previous study, we demonstrated that HA can enter into the central layer of the cartilage tissue, and can support reduction of the mechanical stress-induced ROS [19]. The results of the present study are consistent with the previous results, and suggest that the antioxidation properties of HA are an important biological effect in vitro (cultured cells) [39] and ex vivo (tissues) [19]. However, it is unknown whether the antioxidant effect of HA is derived from physical properties of the HA itself or results from a biosynthetic response of the chondrocytes to the HA.

Therefore, we addressed mechanisms mediating the effect of HA in cultured articular chondrocytes. By adding HA, we found that HA induced accumulation of the critical transcriptional factor Nrf2, and led to corresponding changes in target gene expressions. These results suggested that the effect of HA on the chondrocytes is not due to the chemical properties of the HA, but occurred through biological mechanisms and activation of intracellular signal transduction. Until now, the fact that HA contributes to upregulation of gene expression of the antioxidative stress-related enzymes has not been reported, but there is some prior evidence that HA modulates expression of some genes via its specific receptors CD44 [40,41] or/and RHAMM [42–44].

Furthermore, we tried to elucidate the mechanism how HA induces Nrf2 accumulation by focusing on Akt activity-dependent regulation of the Nrf2 stabilization system. Nrf2 is mainly regulated at the post-transcriptional level. Under normal conditions, Nrf2 is constantly degraded via the ubiquitin–proteasome pathway in a Kelch-like ECH-associated protein (KEAP1)-dependent manner [27,28,31]. In the presence of ROS, the Keap1–Nrf2 connection and subsequent Nrf2 degradation process ceases, Nrf2 is stabilized, and can function as a transcriptional regulator through binding to antioxidant response element (ARE) on the promoter region of the target genes [25,27]. To date, interactions between the Keap1–Nrf2 system and other signaling pathways have been examined in some cell types. For example, some previous studies with cancer cell-lines reported that PI3K/Akt activation is important for the Nrf2 stabilization and activation [45–47]. Wang et al. demonstrated that constitutively active Akt enhanced Nrf2 activity using a retinal pigment epithelia (RPE) cell line [48]. In our study, induction of the Akt phosphorylation by HA addition was observed consistent with findings in other cell types. Furthermore, HA-induced Nrf2 accumulation was blocked by treatment with the Akt inhibitor LY294002 or siRNAs towards HA receptors CD44 and RHAMM. These results suggest that HA affects Nrf2 regulation through interaction with CD44/RHAMM and uses Akt as important intracellular messenger.

In regard to the Nrf2 induction, it is possible to consider two types of mechanisms and consequences. First is a reactive accumulation of Nrf2 towards the ROS generation as described above. Second is a stockpiling against potential risk of ROS [27,46,49,50]. The actual meaning of the latter has not been well understood, but it appears that the steady state activation of Nrf2 provides cytoprotection against oxidant stress. Interestingly, Wruck et al., reported that Nrf2 KO mice had more severe cartilage injuries and more oxidative damage during antibody-induced arthritis (AIA) [51]. It is possible that these mice are susceptible to accumulate oxidative stress damage because of their deficiency in neutralizing ROS.

In developing cartilage of mice, Nrf2 expression is observed in proliferating and pre-hypertrophic chondrocytes in tibia at E15.5, and the expression is maintained in all chondrocyte layers and osteoblasts in cancellous bone after birth [52]. Since normal cartilage is an avascular tissue, chondrocytes basically exist in very hypoxic environment. Once inflammation or vascular invasion occurs, chondrocytes are exposed to enhanced oxidative stresses. Furthermore, compressive mechanical stress induced by excessive exercise or trauma can also be an important source of oxidative stress. Therefore, the previous observation that Nrf2 proteins reside in all layers of cartilage is consistent with the necessities of chondrocyte to protect against the potential risks of oxidative stresses. We also examined here the importance of the Nrf2 expression by comparing the Nrf2 knocked-down chondrocytes with normal chondrocytes. In our experiments, decreased expression of cartilage matrix gene Col2A1, and of the cartilage development related gene Sox9 were observed. In addition, there was reduced expression of genes encoding anti-oxidative stress molecules, and increased gene expression of Adamts-5, an important catabolic molecule for cartilage matrix. Since Nrf2 expression is observed also in normal cartilage development and there is evidence suggesting that Nrf2 contributes to control of some metabolic genes and transcription factors and cell cycle regulators, it is possible that Nrf2 directly regulates cartilage matrix-related gene expression.

Although further evidences including epidemiological studies are essential to link HA levels, Nrf2 stockpile and OA pathogenesis, our results provide an advance in elucidating an important protective mechanism in chondrocyte homeostasis.

## Author contributions

YO and TT wrote the manuscript. TT and KF designed the study and experiments. YO and TT performed the experiments.

## Conflict of interest statement

K.F. received a research Grant from Kaken Pharmaceutical Co., Ltd., Tokyo, Japan.

## Figures and Tables

**Table 1:** Sequences of siRNA duplexes used in the present study.

	Sense	Antisense
Nrf2-1	GUAACUGCAGCCCACAUUUTT	AAAUGUGGGCUGCAGUUACTT
Nrf2-2	GAGACUAGUACAGUUCCAATT	UUGGAACUGUACUAGUCUCTT
Nrf2-3	CAAAGAAAGAAGUACCUGUTT	ACAGGUACUUCUUUCUUUGTT
CD44-1	GGAGAAGAAUGGUCGCUAUTT	AUAGCGACCAUUCUUCUCCTT
CD44-2	CCAGAGAAUACCUCGGAUATT	UAUCCGAGGUAUUCUCUGGTT
CD44-3	CUACAGACUCCUUGGAAAATT	UUUUCCAAGGAGUCUGUAGTT
RHAMM-1	GCAUGUUGUGAAAUUGAAAT	UUUCAAUUUCACAACAUGCTT
RHAMM-2	GCCUUAAGCAGUCUCUUGATT	UCAAGAGACUGCUUAAGGCTT
RHAMM-3	GGUGUUUGAUUAAUAUUUATT	UAAAUAUUAAUCAAACACCTT

**Table 2:** Primers for qPCR used in the present study.

	Forward	Reverse
Sox9	GTACCCGCACCTGCACAAC	CTTGTAATCTGGGTGGTCCTTCTT
Col2A1	TGGTATCGCCGGACCCAAG	CTCGTCCACCGTCCTTCCC
ADAMTS-5	TCACCAGCATTGACGCATC	TGGTAGGTCCAGCAAACAG
HO-1	ACTTTCAGAAGGGTGAGCTGAC	TTGCGTTCGATCTCCTCCTC
NQO-1	TCATCTCCAGAAAGGACATC	ACAGTCTCGGCAGGATACTG
GPX-1	TGCAACCAGTTTGGGCATC	ACGTACTTCAGGCAATTCAGG
Catalase	TCCTGAGAGAGTCGTGCACG	CGTCCTCTTTCCAATATGCTC
Catalase	TCCTGAGAGAGTCGTGCACG	CGTCCTCTTTCCAATATGCTC
SOD-1	TGATCATGGATTCCACGTC	GGACAGAGGATTAAAGTGAGG
Nrf2	CAGCACAACACATACCATCAG	TGCATGCAGTCATCGAAGTAC
GAPDH	GTGAAGGTCGGAGTGAACG	TAAAAGCAGCCCTGGTGAC
